# More powerful parameter tests? No, rather biased parameter estimates. Some reflections on path analysis with weighted composites

**DOI:** 10.3758/s13428-023-02256-5

**Published:** 2023-11-07

**Authors:** Florian Schuberth, Tamara Schamberger, Jörg Henseler

**Affiliations:** 1 Faculty of Engineering Technology, University of Twente, PO Box 217, 7500 AE, Enschede, The Netherlands; 2 Faculty of Business Administration and Economics, Bielefeld University, Universitätsstrasse 25, 33615 Bielefeld, Germany; 3 Nova Information Management School, Universidade Nova de Lisboa, Campus de Campolide, 1070-312 Lisbon, Portugal

**Keywords:** Measurement error, Partial least squares path modeling, PLS-SEM, Effect size, Covariance-based structural equation modeling

## Abstract

Recently, a study compared the effect size and statistical power of covariance-based structural equation modeling (CB-SEM) and path analysis using various types of composite scores (Deng, L., & Yuan, K.-H., *Behavior Research Methods,* *55*, 1460–1479, 2023). This comparison uses nine empirical datasets to estimate eleven models. Based on the meta-comparison, that study concludes that path analysis via weighted composites yields “path coefficients with less relative errors, as reflected by greater effect size and statistical power” (ibidem, p. 1475). In our paper, we object to this central conclusion. We demonstrate that the justification these authors provided for comparing CB-SEM and path analysis via weighted composites is not well grounded. Similarly, we explain that their employed study design, i.e., a meta-comparison, is very limited in its ability to compare the effect size and power delivered across these methods. Finally, we replicated Deng and Yuan’s (ibidem) meta-comparison and show that CB-SEM using the normal-distribution-based maximum likelihood estimator does not necessarily deliver smaller effect sizes than path analysis via composites if a different scaling method is employed for CB-SEM.

## Motivation

Recently, two articles were published that compare covariance-based structural equation modeling (CB-SEM) using the normal-distribution-based maximum likelihood (NML) estimator to regression analysis using various types of weighted composites including partial least squares structural equation modeling (PLS-SEM) (Yuan & Deng, 2021; Yuan & Fang, 2022). In two commentaries, we showed that these articles are mistaken on several points (see, Schuberth et al., 2023; Schuberth, Schamberger, Rönkkö, Liu, & Henseler, 2023). In the meantime, a third paper by Deng and Yuan (2023) was published in this journal following the articles of Yuan and Deng (2021) and Yuan and Fang (2022). In this study, Deng and Yuan contrast CB-SEM using the NML estimator with path analysis using different types of composite scores. The latter comprises path analysis using Bartlett factor scores (BFS, Bartlett, 1937), equally weighted composite (EWC) scores, PLS-SEM Mode A composite scores (Wold, 1982), and PLS-SEM Mode $\text {B}_{\text {A}}$ composite scores, which was recently introduced by Yuan and Deng (2021). Assuming that the estimates of path analysis using composite scores differ from CB-SEM estimates only in terms of a scaling factor, the Deng and Yuan (2023) paper aims to compare the statistical power these methods delivered. In doing so, this paper considers the signal-to-noise ratio, an effect size measure proposed by Yuan and Fang (2022). To this end, Deng and Yuan conducted a meta-comparison in which they studied 11 empirical examples with 47 path coefficient estimates and, thus, 47 empirical effect sizes. The results of the meta-comparison indicate that PLS-SEM using Mode $\text {B}_{\text {A}}$ most often produces estimates with the largest absolute effect sizes and yields the largest average absolute effect size, whereas CB-SEM delivers the smallest average absolute effect size. Based on these findings, the paper concludes that path analysis via composites yields “path coefficients with less relative errors, as reflected by greater effect size and statistical power” (Deng & Yuan 2023, p. 1475).

Unfortunately, the central conclusion of the Deng and Yuan (2023) paper is not tenable. In the following three sections, we point out three issues in Deng and Yuan (2023)’s work. These issues lead Deng and Yuan (2023) to a conclusion that is not generally true. First, the Deng and Yuan (2023) paper draws heavily on the assertion that identical values for the path coefficients under CB-SEM and path analysis with factor-wise composite scores can always be obtained by adjusting the scales of the latent variables or the composites, respectively. However, as we demonstrate analytically and by means of a counterexample, the assertion is not generally true and applies only under exceptional circumstances. Hence, the parameter values of path analysis with factor-wise composite scores cannot always be transformed into the values obtained by CB-SEM by adjusting the scales of the composites and vice versa. Second, the study design the Deng and Yuan (2023) paper reports does not allow us to draw informed conclusions about the effect size and power delivered by the different methods because it is based on empirical data for which we know neither the true underlying mechanisms, nor the true effects. Specifically, while the Deng and Yuan (2023) paper claims to study the statistical power of methods (i.e., the conditional probability of detecting an effect under the condition that the effect really exists), it actually only studies the relative frequency of signaling an effect, independent of whether the effect really exists. Third, and as is also done in the Yuan and Fang (2022) study, the Deng and Yuan (2023) paper misses to mention that the empirical signal-to-noise ratio under CB-SEM using NML, which is used as empirical effect size measure, depends on which method is used to determine the scale of the latent variables. Reconstructing the meta-comparison Deng and Yuan (2023) conducted, we find that when a different scaling method is used for CB-SEM, the results change considerably and no longer support the conclusions drawn in the Deng and Yuan (2023) paper.

Against this background, we conclude that empirical studies modeling relationships between latent variables will not benefit from relying on path analysis with composite scores: While one cannot be certain whether this methodological choice will result in a gain or loss of statistical power in a particular research situation, one can be almost certain that the analysis will suffer from biased estimates and inflated type I error rates.

## Scaling cannot correct for attenuation bias

In their study, Deng and Yuan (2023) considered path analysis with factor-wise composite scores, i.e., with the scores of a latent variable computed exclusively based on its connected indicators. It is widely known in the literature, and also acknowledged by Deng and Yuan (2023), that path analysis using factor-wise composite scores produces biased estimates due to attenuation which is caused by the random measurement error comprised in the composite scores (e.g., Dijkstra, 1985; Bollen, 1989; Lu, Kwan, Thomas, & Cedzynski, 2011; Dijkstra & Henseler, 2015a; Yuan, Wen, & Tang, 2020; Devlieger, Mayer, & Rosseel, 2016; Croon, 2002; Skrondal & Laake, 2001; Schuberth et al., 2023). However, to justify the comparison of the effect sizes and power between CB-SEM and path analysis via composites, Deng and Yuan (2023, p.1461) assume that “for a set of given values of the path coefficients among latent variables, one can obtain identical values for these coefficients under path analysis with composite scores by adjusting the scales of the composites”.

We demonstrate that Deng and Yuan ’s (2023) assumption is not generally true and that attenuation bias in path analysis via composites cannot always be corrected by adjusting the scales of the composites. To do so, we consider a single regression equation containing one dependent latent variable $\eta _\text {dep}$ and *k* independent latent variables $\varvec{\eta }_\text {ind}$. Both the dependent variable and the independent latent variables are each reflectively measured by a set of indicators: $\varvec{x}_\text {dep} = \varvec{\lambda }_\text {dep} \eta _\text {dep}+ \varvec{\varepsilon }_\text {dep}$ and $\varvec{x}_{\text {ind},j} = \varvec{\lambda }_{\text {ind},j} \eta _{\text {ind},j} +\varvec{\varepsilon }_{\text {ind},j}$, $j=1,...,k$, where the measurement errors $\varepsilon$ are assumed to have a mean of zero and are uncorrelated among each other. Further, each indicator is assumed to load only on one latent variable and each latent variable is assumed to have a mean of zero. This leads to the following regression equation:

1 $$\begin{aligned} \eta _\text {dep}= \varvec{\gamma }' \varvec{\eta }_\text {ind} + \zeta \end{aligned}$$

where $\varvec{\gamma }$ is the vector containing the regression coefficients among the latent variables and $\zeta$ depicts the structural disturbance term, which is assumed to be uncorrelated with the independent latent variables and the random measurement errors $\varepsilon$.

Deng and Yuan (2023) considered factor-wise composite scores, i.e., they calculated the composite scores for a latent variable based on its directly connected indicators. In this case, each independent latent variable $\eta _{\text {ind},j}$ is replaced by a composite, i.e., $\tilde{\eta }_{\text {ind},j}=\varvec{w}_{\text {ind},j}'\varvec{x}_{\text {ind},j}=\varvec{w}_{\text {ind},j}' (\varvec{\lambda }_{\text {ind},j} \eta _{\text {ind},j} + \varvec{\varepsilon }_{\text {ind},j})= \varvec{w}_{\text {ind},j}'\varvec{\lambda }_{\text {ind},j} \eta _{\text {ind},j} + \varvec{w}_{\text {ind},j}' \varvec{\varepsilon }_{\text {ind},j}=q_{\text {ind},j} \eta _{\text {ind},j} + \delta _{\text {ind},j}$. The same applies to the dependent latent variable: $\tilde{\eta }_\text {dep}=\varvec{w}_\text {dep}'\varvec{\lambda }\eta _\text {dep} + \varvec{w}_\text {dep}'\varvec{\varepsilon }_\text {dep} = q_\text {dep} \eta _\text {dep} + \delta _\text {dep}$. Consequently, the composite scores for the dependent and independent latent variables are contaminated by random measurement error. As shown in the Appendix, the parameter estimates $\hat{\tilde{\varvec{\gamma }}}$ of a regression using factor-wise composite scores converge in probability to:

2 $$\begin{aligned} \mathop {\textrm{plim}}\limits \hat{\tilde{\varvec{\gamma }}} = (\varvec{Q} \varvec{\Sigma }\varvec{Q} + \varvec{\Sigma }_\delta )^{-1} \varvec{Q} \varvec{\Sigma }q_\text {dep} \varvec{\gamma } \end{aligned}$$

where $\varvec{\Sigma }$ is the true variance-covariance matrix of the independent latent variables $\eta _{\text {ind},j}$, and $\varvec{\Sigma }_\delta$ the variance-covariance matrix of the corresponding composed random measurement error terms $\delta _{\text {ind},j}$. Note that the latter is a diagonal matrix because the measurement errors $\varepsilon$ are mutually uncorrelated. Similarly, $\varvec{Q}$ is a diagonal matrix containing the *q*s of the composites used for the independent latent variables, i.e., $q_{\text {ind},j}$. Note, the biasing factor of the regression coefficients $(\varvec{Q} \varvec{\Sigma }\varvec{Q} + \varvec{\Sigma }_\delta )^{-1} \varvec{Q} \varvec{\Sigma }q_\text {dep}$ resembles the reliability matrix known from the error-in-variables literature (e.g., Gleser, 1992).

Considering Eq. 2, it can be seen that, in principle, the probability limit of the estimates $\hat{\varvec{\gamma }}$ of a regression using factor-wise composite scores can be transformed into the population parameters $\varvec{\gamma }$. However, it is emphasized that this bias cannot generally be corrected by adjusting the scales of the composites, i.e., multiplying a composite with a constant because $(\varvec{Q} \varvec{\Sigma }\varvec{Q} + \varvec{\Sigma }_\delta )^{-1} \varvec{Q} \varvec{\Sigma }q_\text {dep}$ is not necessarily a diagonal matrix. Hence, a diagonal matrix that can be multiplied with the biasing factor to obtain a unit matrix, does not always exist. Exemptions are situations in which all independent latent variables are uncorrelated, and thus, $\varvec{\Sigma }$ equals a diagonal matrix, or the case of a simple regression, i.e., a regression equation with only one independent latent variable. Note that Deng and Yuan ’s (2023, Footnote 3) study did consider a simple regression model case to demonstrate that “one can always make the composites estimates unbiased by rescaling the composites”. Consequently, it is not generally possible to obtain identical values for the path coefficients under path analysis with composite scores as under CB-SEM by adjusting the scales of the composites (or latent variables).

**Fig. 1:**
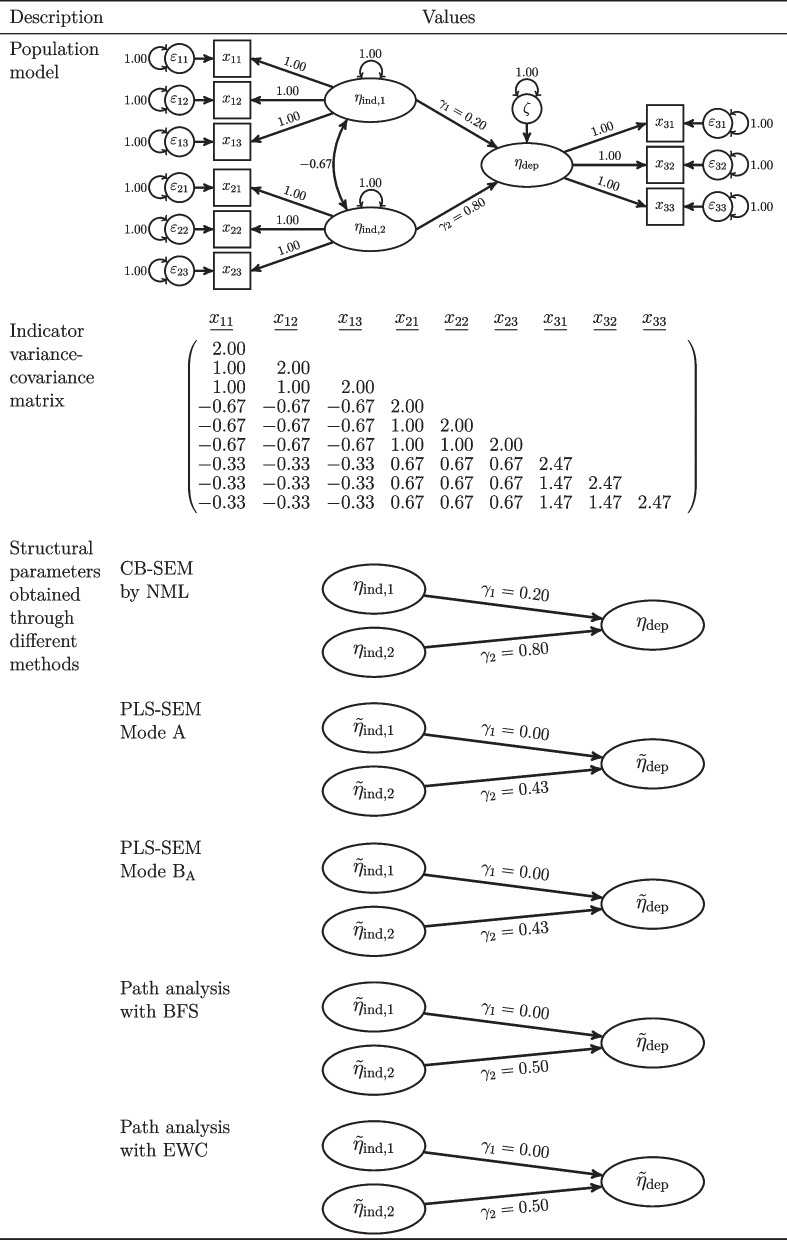
Illustrative counterexample. Note: Values are rounded to the second decimal. CB-SEM: covariance-based structural equation modeling; NML: normal-distribution-based maximum likelihood; PLS-SEM partial least squares structural equation modeling; BFS: Bartlett factor scores; EWC: equally weighted composites

To provide an illustrative counterexample for Deng and Yuan ’s (2023) claim, we focus on a model containing one dependent latent variable that is predicted by two independent latent variables:

3 $$\begin{aligned} \eta _\text {dep} = \gamma _1 \eta _{\text {ind},1} + \gamma _2 \eta _{\text {ind},2} + \zeta \end{aligned}$$

where the two independent latent variables show a mean of 0, a variance of 1, and a covariance of -2/3. Similarly, the disturbance term $\zeta$ is uncorrelated with all exogenous variables and shows a unit variance. Further, each latent variable is measured by three indicators, each with a factor loading of 1. The corresponding random measurement errors are uncorrelated and their variances are set to 1. Consequently, each latent variable is measured by a set of parallel measures (Lord & Novick, 2008). The complete population model and its variance-covariance matrix are illustrated in Fig. 1. A similar example was given in Schuberth et al. (2023) to show that it is not generally true that a structural parameter under PLS-SEM is equal to 0 if and only if the corresponding structural parameter equals zero under CB-SEM as claimed by Yuan and Deng (2021).

In a next step, we apply CB-SEM and path analysis using the four types of composite scores considered by Deng and Yuan (2023). To obtain the CB-SEM parameters, we apply the NML estimator as implemented in the R package lavaan (Rosseel, 2012). To calculate the BFS, we use the CB-SEM results. Considering PLS-SEM using Mode A and Mode B_A_, we use the R package matrixpls (Rönkkö, 2022), which allows us to run the PLS algorithm on the indicator variance-covariance matrix as Deng and Yuan (2023) have done, instead of the indicator correlation matrix.^1^

Figure 1 shows the results for the different methods. The results highlight that it is not possible to adjust the latent variables’ scales to draw a coefficient different from zero under CB-SEM, i.e., $\gamma _1$, into a coefficient of zero as obtained by path analysis with composite scores. Similarly, it is not possible to adjust the composites’ scales to obtain a value different from 0 for $\gamma _1$ under path analysis via composites. Consequently, the values of the path estimates under path analysis via composites cannot in general be transferred into the CB-SEM value of this parameter by adjusting the scales of the composites, and vice versa.

*Conclusion:* Deng and Yuan ’s (2023) central assumption, which they use for justifying the comparison of CB-SEM and path analysis using composite scores, is wrong. Usually, it is not possible to correct the attenuation bias present in path analysis using factor-wise composite scores by adjusting the scales of the composites. Consequently, effect size measures and, thus, significance tests on individual parameters and their power can be biased and misleading under path analysis with composite scores even if the CB-SEM model is correctly specified.

## Unsuitable study design

To answer their research question, i.e., “Which method is more powerful in testing the relationship of theoretical constructs?” Deng and Yuan (2023) conducted a meta-comparison. In this meta-comparison, they considered nine datasets to estimate 11 models comprising three to six latent variables. For the estimation, they used five different methods including CB-SEM using NML and path analysis using various types of composite scores including BFS, EWC scores, PLS-SEM Mode A composite scores, and PLS-SEM Mode B_A_ composite scores. To account for non-normality in the studied datasets, they used M-estimates of the means and covariances to estimate the model parameters. This was only done if the dataset showed a potential violation of the multivariate normality assumption and if the dataset and not only its variance-covariance matrix was available. For more details on the study design and the robust transformation to account for non-normality, the reader is referred to the original study of Deng and Yuan (2023). Finally, the empirical effect sizes were compared across methods to provide an answer to their research question.

By using a meta-comparison, Deng and Yuan (2023) implicitly assume that this research design is suitable for answering their research question, but they do not provide arguments for why this research design should be valid, nor do they cite other research that demonstrates that meta-comparisons are a viable means of comparing the behavior of different methods, in particular their delivered effect size and statistical power. We would argue that meta-comparisons are an inappropriate study design for this purpose. The meta-comparison conducted by Deng and Yuan (2023) provides answers as to which method most often shows the largest effect size. However, it does not allow us to draw informed conclusions about a method’s delivered effect size and power in testing the significance of path coefficients. The effect size is “the degree to which the null hypothesis is false” (Cohen, 1988, pp.9). Similarly. the power of a statistical test is defined as the probability that a statistical test will reject a null hypothesis *if it is indeed false* (e.g., Cohen, 1992). This means that statistical power is a conditional probability, with the condition that the null hypothesis is false. The null hypothesis is a statement about a population parameter (Casella & Berger, 2001, Chapter 8), which is (typically) unknown in empirical studies. Therefore, evaluating a method’s delivered effect size and statistical power by means of empirical data is a difficult endeavor.

**Fig. 2:**
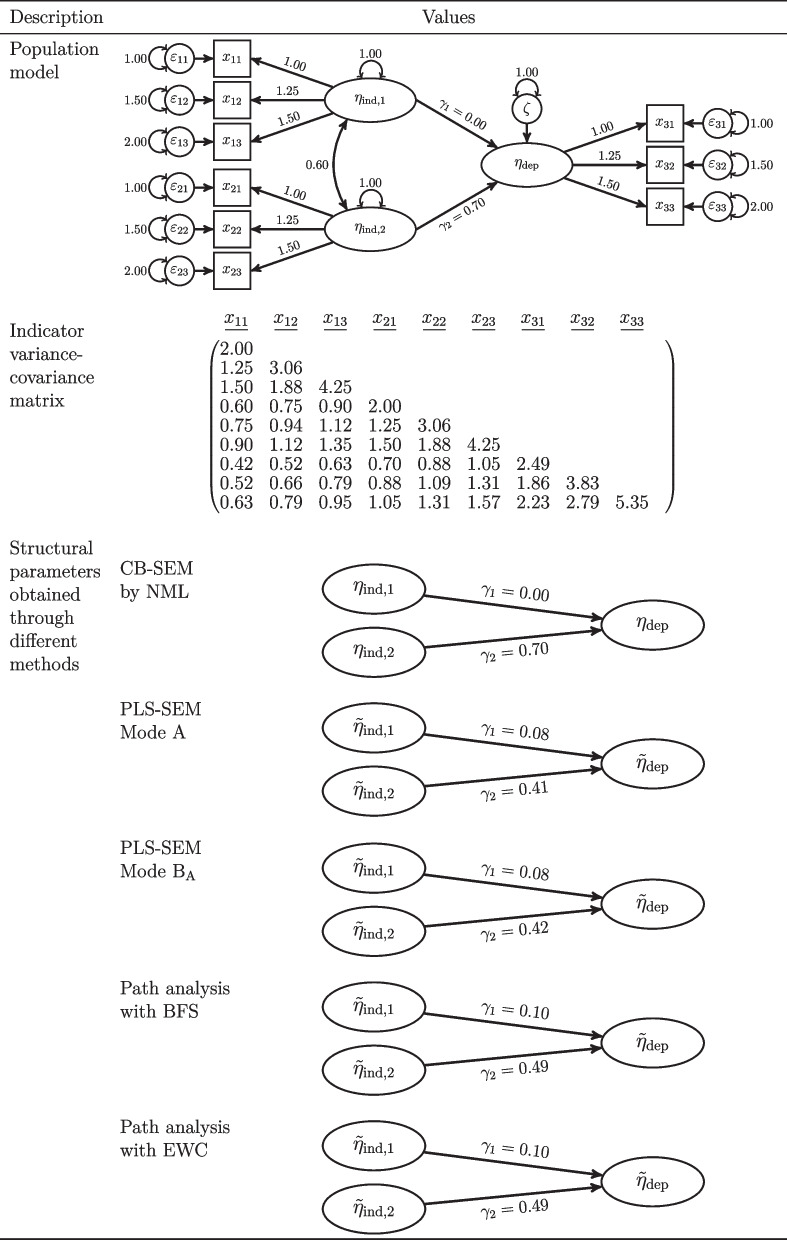
Illustrative example. Note: Values are rounded to the second decimal. CB-SEM covariance-based structural equation modeling, NML normal-distribution-based maximum likelihood, PLS-SEM partial least squares structural equation modeling, BFS Bartlett factor scores, EWC equally weighted composites

To illustrate this problem, we consider an example similar to the one used in the commentary of Schuberth et al. (2023, Table 1).^2^ The population model in Fig. 2 illustrates that in this example the true effect of $\eta _{\text {ind},1}$ on $\eta _\text {dep}$ is zero, i.e., $\gamma _1=0$. Under CB-SEM this effect is correctly estimated at zero, thus the effect size will equal zero, whereas under path analysis via composites this path coefficient is other than zero, thus the size of this effect will also be other than zero. Following Deng and Yuan ’s (2023) reasoning, one would conclude for this effect that CB-SEM delivers the smallest effect size and power of all the considered methods. However, this should not be interpreted as an advantage path analysis via composites has over CB-SEM. In fact, path analysis using composite scores signals an effect that is not present. Similarly, this is an indication of an inflated type I error rate under path analysis via composites since H_0_ is not false in this case. The error-in-variables literature studied a very similar case and conclude that researchers “almost sure commit a Type I error” (Brunner & Austin, 2009, p. 37). This is also highlighted in the PLS-SEM literature which provides “strong evidence that [PLS-SEM] detects an unacceptably high number of ‘false positive”’ (Goodhue, Lewis, & Thompson, 2006, p.8). A further example is provided in Fig. 1. In this case, $H_0$ is false and path analysis via composites would wrongly deliver an effect size of 0 for the effect of $\eta _{\text {ind},1}$ on $\eta _\text {dep}$. This effect size is also smaller than the one delivered by CB-SEM.

**Table 1:** Counts for each method to yield the largest and smallest absolute *z*-statistics, as well as the mean rank (1 to 5) and mean value of the absolute *z*-statistics across the 41 parameters using the scaling method for CB-SEM employed in Deng and Yuan ’s (2023) study

	CB-SEM by NML	Path analysis with BFS	Path analysis with EWC	PLS-SEM Mode A	PLS-SEM Mode B_A_
Count of largest |*z*|	2	10	5	5	19
Count of smallest |*z*|	34	0	6	0	1
Average rank of |*z*|	1.39	3.46	2.89	3.35	3.90
Average value of |*z*|	6.61	8.16	8.00	8.08	8.22

Note: CB-SEM: covariance-based structural equation modeling; NML: normal-distribution-based maximum likelihood; BFS: Bartlett factor scores; EWC: equally weighted composites; PLS-SEM: partial least squares structural equation modeling. For CB-SEM by NML, the variance of the exogenous latent variables and one loading for each endogenous latent variable are fixed to 1

As can be seen from these two examples, it is rather difficult to come up with general conclusions about the performance of path analysis via composites in terms of effect size and power delivered. This is because its estimates are biased due to attenuation, which can likely not be corrected by adjusting the composites’ scales in case of models with more than two latent variables. For exemptions, the reader is referred to the previous section. Consequently, the effect sizes under path analysis via composites are also likely distorted. Because the direction of the attenuation bias and the true effects are unknown in empirical studies, assessing effect size and power in path analysis via composites by meta-comparison is quite difficult, if not impossible. This also highlights the importance of not considering the statistical power of a method in isolation, but taking into account the type I error rate. Otherwise, flawed methods that always reject the null hypothesis of no effect, regardless of the true effect size, and thus have a statistical power of 100%, would be mistakenly preferred over existing methods that maintain the predefined significance level.

A more promising alternative that Deng and Yuan (2023) also mention for investigating a method’s delivered effect size and power, is Monte Carlo simulations, which allow us to determine all the conditions under which a method is studied (e.g., Paxton, Curran, Bollen, Kirby, & Chen, 2001; Schamberger, 2023). Note that empirical datasets are typically studied to improve our understanding of given mechanisms in the world, while Monte Carlo simulations are usually conducted to improve our understanding of a method (Goodhue, Lewis, & Thompson, 2012).

*Conclusion:* Deng and Yuan ’s (2023) study provides only very limited insights on which method delivers a larger effect size and power since the mechanisms including true effects underlying a dataset are unknown in empirical studies. For empirical examples, it is not clear whether findings about a method are due to the method’s performance or due to peculiarities of the studied dataset.

## The empirical signal-to-noise ratio depends on the scaling method under CB-SEM

Deng and Yuan ’s (2023) study compares CB-SEM using NML to path analysis with various types of composite scores regarding their delivered effect sizes and power. For path analysis via composites, they considered four different types of composite scores, i.e., BFS, EWC, and composite scores created by PLS-SEM Mode A and PLS-SEM Mode B_A_. As effect size measure, their study proposed using the signal-to-noise-ratio (see also Yuan & Fang, 2022), which is defined as $\tau _\gamma =E(\hat{\gamma })/[N \text {Var}(\hat{\gamma })]^{1/2}$, where $\hat{\gamma }$ is the regression coefficient, i.e., the estimated effect, and *N* equals the sample size. Hence, the empirical signal-to-noise ratio corresponding to a regression coefficient $\hat{\gamma }$ equals the *z*-statistic of this coefficient divided by $\sqrt{N}$.^3^

We know from the CB-SEM literature that the value of the *z*-statistic depends on the method used to fix the scales of the latent variables if NML is used for model estimation (e.g., Gonzalez & Griffin, 2001; Klopp & Klößner, 2021). Consequently, the empirical signal-to-noise ratio also depends on the employed scaling method (Schuberth, et al., 2023).

To demonstrate this issue, we reconsider Deng and Yuan ’s (2023) meta-comparison. In contrast to the original study, we considered only models for which the dataset or the variance-covariance matrix was freely available, i.e., all datasets except Dataset 7. Consequently, we focus on ten models, instead of 11 models, using eight different datasets, which results in 41 path coefficient estimates. For more details on the models and the used datasets, the reader is referred to Deng and Yuan (2023). Note that instead of considering the empirical signal-to-noise ratio directly, we follow Deng and Yuan (2023) and focus on the *z*-statistics. To obtain the CB-SEM estimates, in Deng and Yuan ’s (2023) study the variance of the exogenous latent variables and one loading of each endogenous latent variable are fixed to 1 to determine the latent variables’ scales. The BFS are calculated using the CB-SEM parameter estimates, while for PLS-SEM, the weights are chosen in such a way that the composites show a unit variance. Table 1 summarizes the results of the ten models.

**Table 2:** Counts for each method to yield the largest and smallest absolute *z*-statistics, as well as the mean rank (1 to 5) and mean value of the absolute *z*-statistics across the 41 parameters using a scaling method for CB-SEM different to the one employed in Deng and Yuan ’s (2023) study

	CB-SEM by NML	Path analysis with BFS	Path analysis with EWC	PLS-SEM Mode A	PLS-SEM Mode B_A_
Count of largest |*z*|	21	4	4	5	7
Count of smallest |*z*|	18	5	16	0	2
Average rank of |*z*|	3.12	3	2.48	2.91	3.49
Average value of |*z*|	10.82	8.16	8.00	8.08	8.22

Note: CB-SEM: covariance-based structural equation modeling; NML: normal-distribution-based maximum likelihood; BFS: Bartlett factor scores; EWC: equally weighted composites; PLS-SEM: partial least squares structural equation modeling. For CB-SEM by NML, the variance of each latent variable is fixed to 1

The results in Table 1 are very similar to those reported in Deng and Yuan ’s (2023) Table 12, i.e., the results based on all 11 models. Deng and Yuan (2023, p. 1475, footnote omitted) summarize these results as indicating “that CB-SEM has the smallest power and/or effect size in testing the significance of the path coefficients of the structural models. In contrast, PLS-SEM mode B_A_ yielded the largest average *z*-statistics and average rank, followed by path analysis with Bartlett-factor scores.” Based on these findings Deng and Yuan (2023, p.1475) conclude that path analysis via weighted composites yields “path coefficients with less relative errors, as reflected by greater effect size and statistical power.”

To demonstrate the impact of the scaling method on the results of Deng and Yuan (2023) and their conclusion, we re-estimate the ten models by CB-SEM using a different scaling method. Little (2013) emphasizes that the specific choice of scaling is arbitrary: While at least one parameter must be fixed for each latent variable, it does not matter which one is fixed. “Different methods of scaling result in mathematically equivalent models that show identical overall model fit and give the same answers to key questions” (Geiser, 2021, p. 120). For our analysis, we choose an alternative scaling method that has the elegant feature of providing standardized coefficients. This scaling method was for instance implemented in the SEM software RAMONA (Browne & Mels, 1992). As suggested by Kwan and Chan (2011) as one way to achieve this, we set nonlinear constraints on the variances of the structural disturbance terms in a way that implies that the variances of the corresponding endogenous latent variables are equal to 1. In addition, we fix the variances of the exogenous latent variables to 1. The same scaling was applied in Schuberth et al. (2023). Note that the scaling method used for CB-SEM does not affect the signal-to-noise ratio under path analysis using BFS. Table 2 juxtaposes these CB-SEM results with those of path analysis using composite scores.^4^

The results of Table 2 differ from those reported in Table 1. Using a different scaling method, CB-SEM yields the largest average absolute *z*-statistics and, thus, the largest average absolute empirical signal-to-noise ratio for CB-SEM among the five considered methods. In addition, for more than 50% of the 41 considered path coefficients, CB-SEM produces the largest absolute *z*-statistics.

*Conclusion:* The empirical signal-to-noise ratio, which is a transformation of the *z*-statistic, depends under CB-SEM using NML on the scaling method employed. Re-analyzing ten of the 11 models (Deng & Yuan, 2023) studied and using a different scaling method for CB-SEM, the results do not support Deng and Yuan ’s (2023) conclusion. In fact, of all the five considered methods, CB-SEM produced the largest average absolute value of the *z*-statistic.

## Discussion and conclusion

Based on a meta-comparison of eleven models using nine datasets, Deng and Yuan (2023, p. 1475) conclude that “path analysis via weighted composites has an additional advantage of yielding path coefficients with less relative errors, as reflected by greater effect size and statistical power”. In our commentary, we raise objections to this conclusion.

It might appear surprising that we object to Deng and Yuan ’s conclusion; actually more than ten years ago in a co-authored Monte Carlo simulation study, the last author of this commentary also concluded himself that “the statistical power of [PLS-SEM] is always larger than or equal to that of CBSEM” (Reinartz, Haenlein, & Henseler, 2009, Abstract). However, since then, lots of research has been devoted to this paradoxical phenomenon (cf. Dijkstra & Henseler, 2015b; Goodhue, Lewis, & Thompson, 2017; Rönkkö & Evermann, 2013), and it is now much better understood. As a result of the research endeavors of the last decade, it is now known that the presumably higher statistical power of PLS-SEM and other composite-based methods is spurious. It is a methodological artifact resulting from attenuation through random measurement error combined with multicollinearity (Goodhue et al., 2017). As a reaction, Henseler (2020, p. 86) explicitly states that Reinartz et al. (2009) “should be read with caution,” and Benitez, Henseler, Castillo, and Schuberth (2020, p. 5) explain that the findings of Reinartz et al. (2009) with regard to statistical power “are highly questionable, as they are based on [PLS-SEM], which is known to produce inconsistent parameter estimates for latent variable models. In line with [Goodhue et al. (2017)], who show that this alleged higher power goes along with an inflated type I error, we conclude that preferring [PLS-SEM] over the [NML] estimator due to efficiency is not a valid argument for latent variable models.”

In our commentary, we provide three reasons why the conclusion of Deng and Yuan ’s (2023) paper is based on a weak foundation and eventually is incorrect. First, we demonstrate that bias under path analysis with composite scores is substantive and cannot be corrected by adjusting the scales of the composites. Hence, effect size measures and power are most likely distorted under path analysis via composites even though the model is correctly specified. Consequently, methods that take into account random measurement error such as CB-SEM, consistent PLS (Dijkstra & Henseler, 2015b) and factor score regression with a correction for attenuation (e.g., Devlieger et al., 2016; Lu et al., 2011; Rosseel & Loh, 2022; Yuan et al., 2020), should be preferred over path analysis via composites when it comes to testing relationships between theoretical constructs. Second, Deng and Yuan ’s (2023) meta-comparison of empirical studies is very limited in its ability to draw informed conclusions about the effect size and power delivered by a method. This is because in empirical datasets the true effect size is unknown. Third, we show that Deng and Yuan ’s (2023) findings depend largely on the scaling method used for CB-SEM estimated by NML. If a scaling method different to the one used in Deng and Yuan ’s (2023) study is employed, the results do not necessarily support their findings. Against this background, the conclusion of Deng and Yuan ’s (2023 p. 1475) study that path analysis via composites yields “path coefficients with less relative errors, as reflected by greater effect size and statistical power”, is not tenable.

## Data Availability

The complete R code can be downloaded from the following URL: https://osf.io/ym3xt/?view_only=5dbd179234de42bcbfecc2f5396dc239.

## Appendix

### Proof: Probability limit of path analysis using factor-wise composite scores

Starting point is one equation of a recursive structural model connecting a dependent latent variable $\eta _\text {dep}$ to *k* latent predictor variables $\varvec{\eta }_\text {ind}$:

4 $$\begin{aligned} \eta _\text {dep} = \varvec{\gamma }' \varvec{\eta }_\text {ind} + \zeta \end{aligned}$$

Assuming that the structural disturbance term $\zeta$ is uncorrelated with the predictors, the true structural coefficients are obtained as follows:

5 $$\begin{aligned} \varvec{\gamma }&= \varvec{\Sigma }^{-1} \varvec{\Sigma }_{\varvec{\eta }_\text {ind}\varvec{\eta }_\text {dep}} \Leftrightarrow \end{aligned}$$

6 $$\begin{aligned} \varvec{\Sigma }_{\varvec{\eta }_\text {ind}\varvec{\eta }_\text {dep}}&= \varvec{\Sigma }\varvec{\gamma } \end{aligned}$$

where $\varvec{\Sigma }$ is the true variance-covariance of the independent latent variables and $\varvec{\Sigma }_{\varvec{\eta }_\text {ind}\varvec{\eta }_\text {dep}}$ is the vector containing the true covariances between the independent and dependent latent variables.

As explained, in path analysis via factor-wise composites, the composite $\tilde{\eta }_{\text {ind},j}$ for each independent latent variable is obtained as follows:

7 $$\begin{aligned} \tilde{\eta }_{\text {ind},j}=\varvec{w}_{\text {ind},j}'\varvec{x}_{\text {ind},j} = \varvec{w}_{\text {ind},j}'\varvec{\lambda }_{\text {ind},j} \eta _{\text {ind},j} + \nonumber \\ \varvec{w}_{\text {ind},j}'\varvec{\varepsilon }_{\text {ind},j} =q_{\text {ind},j}\eta _{\text {ind},j} + \delta _{\text {ind},j}, \forall j=1,...,k. \end{aligned}$$

The same applies for the dependent latent variable, i.e., $\tilde{\eta }_\text {dep}=\varvec{w}_\text {dep}'\varvec{\lambda }_\text {dep} \eta _\text {dep}+\varvec{w}_\text {dep}' \varvec{\varepsilon }_\text {dep}=q_\text {dep} \eta _\text {dep}+\delta _\text {dep}$. Replacing the independent and dependent latent variables of Eq. 4 by their corresponding composite scores, the least squares (LS) estimates are obtained as follows:

8 $$\begin{aligned} \hat{\tilde{\varvec{\gamma }}}=\varvec{S}^{-1}_{\tilde{\varvec{\eta }}_\text {ind}\tilde{\varvec{\eta }}_\text {ind}}\varvec{S}_{\tilde{\varvec{\eta }}_\text {ind}\tilde{\eta }_\text {dep}}, \end{aligned}$$

where $\varvec{S}_{\tilde{\varvec{\eta }}_\text {ind}\tilde{\varvec{\eta }}_\text {ind}}$ equals the sample variance-covariance matrix of the scores for the independent latent variables and the column vector $\varvec{S}_{\tilde{\varvec{\eta }}_\text {ind}\tilde{\varvec{\eta }}_\text {dep}}$ contains the sample covariances between the composites for the independent and dependent latent variables.

Consequently, these LS estimates converge in probability to the following expression:

9 $$\begin{aligned} \mathop {\textrm{plim}}\limits \hat{\tilde{\varvec{\gamma }}} = \varvec{\Sigma }_{\tilde{\varvec{\eta }}_\text {ind}\tilde{\varvec{\eta }}_\text {ind}}^{-1}\varvec{\Sigma }_{\tilde{\varvec{\eta }}_\text {ind}\tilde{\eta }_\text {dep}}, \end{aligned}$$

where $\varvec{\Sigma }_{\tilde{\varvec{\eta }}_\text {ind}\tilde{\varvec{\eta }}_\text {ind}}$ is the true variance-covariance matrix of the composites used for the independent latent variables and the column vector $\varvec{\Sigma }_{\tilde{\varvec{\eta }}_\text {ind}\tilde{\eta }_\text {dep}}$ contains the true covariances between the composites used for the independent and dependent latent variables.

From Eq. 7 it is apparent that

10 $$\begin{aligned} \varvec{\Sigma }_{\tilde{\varvec{\eta }}_\text {ind}\tilde{\varvec{\eta }}_\text {ind}} = \varvec{Q} \varvec{\Sigma }\varvec{Q} + \varvec{\Sigma }_{\delta }, \end{aligned}$$

where $\varvec{Q}$ is a diagonal matrix containing the *q*s for the independent latent variables, i.e., $q_{\text {ind},j}$, and $\varvec{\Sigma }_\delta$ is the true variance-covariance matrix of the corresponding composed random measurement error terms, i.e., $\delta _{\text {ind},j}$. Using Eq. 6, the true covariance matrix $\varvec{\Sigma }_{\tilde{\eta }_\text {ind}\tilde{\eta }_\text {dep}}$ is calculated as follows:

11 $$\begin{aligned} \varvec{\Sigma }_{\tilde{\varvec{\eta }}_\text {ind}\tilde{\eta }_\text {dep}} =\varvec{Q} \varvec{\Sigma }_{\varvec{\eta }_\text {ind}\eta _\text {dep}} q_\text {dep}=\varvec{Q} \varvec{\Sigma }\varvec{\gamma }q_\text {dep} \end{aligned}$$

Consequently, the estimates of a regression analysis using factor-wise composite scores converge in probability to:

12 $$\begin{aligned} \text {plim}~\hat{\tilde{\varvec{\gamma }}}= \varvec{(}\varvec{Q} \varvec{\Sigma }\varvec{Q} + \varvec{\Sigma }_\delta )^{-1}\varvec{Q} \varvec{\Sigma }q_{\text {dep}} \varvec{\gamma }\end{aligned}$$

## Footnotes

1 The complete R code can be downloaded from the following url: https://osf.io/ym3xt/?view_only=97329a6f461a47e6aaf83dc46c4e27fe

2 The complete R code can be downloaded from the following url: https://osf.io/ym3xt/?view_only=97329a6f461a47e6aaf83dc46c4e27fe

3 We thank a reviewer who pointed out that the signal-to-noise ratio may not even qualify as an effect size measure because it is proportional to the *z*-statistic. For a more detailed explanation, the reader is referred to Rönkkö, Lee, Evermann, McIntosh, and Antonakis (2023).

4 The data and the corresponding model specifications to replicate the results are available at https://osf.io/ym3xt/?view_only=97329a6f461a47e6aaf83dc46c4e27fe.
